# Health literacy among different age groups in Germany: results of a cross-sectional survey

**DOI:** 10.1186/s12889-016-3810-6

**Published:** 2016-11-09

**Authors:** Eva-Maria Berens, Dominique Vogt, Melanie Messer, Klaus Hurrelmann, Doris Schaeffer

**Affiliations:** 1 Department of Health Services Research and Nursing Science, School of Public Health, Bielefeld University, Universitaetsstrasse 25, 33615 Bielefeld, North-Rhine Westphalia Germany; 2 Hertie School of Governance, Friedrichstraße 180, 10117 Berlin, Germany

**Keywords:** Health literacy, Socio-economic factors, Doctor visits, Germany, General population, Age groups, Survey, HLS-EU-Q

## Abstract

**Background:**

Health literacy is of increasing importance in public health research. It is a necessary pre-condition for the involvement in decisions about health and health care and related to health outcomes. Knowledge about limited health literacy in different age groups is crucial to better target public health interventions for subgroups of the population. However, little is known about health literacy in Germany. The study therefore assesses the prevalence of limited health literacy and associated factors among different age groups.

**Methods:**

The Health Literacy Survey Germany is a cross-sectional study with 2,000 participants aged 15 years or older in private households. Perceived health literacy was assessed via computer-assisted personal interviews using the HLS-EU-Q-47 questionnaire. Descriptive analyses, chi-square tests and odds ratios were performed stratified for different age groups.

**Results:**

The population affected by limited perceived health literacy increases by age. Of the respondents aged 15–29 years, 47.3 % had limited perceived health literacy and 47.2 % of those aged 30–45 years, whereas 55.2 % of the respondents aged 46–64 years and 66.4 % aged 65 years and older showed limited perceived health literacy. In all age groups, limited perceived health literacy was associated with limited functional health literacy, low social status, and a high frequency of doctor visits.

**Conclusions:**

The results suggest a need to further investigate perceived health literacy in all phases of the life-course. Particular attention should be devoted to persons with lower social status, limited functional health literacy and/or a high number of doctor visits in all age groups.

## Background

Health literacy is the competence to access, understand, appraise, and apply health information in order to take decisions in everyday life concerning healthcare, disease prevention, and health promotion [1]. This definition goes beyond functional literacy [2]. Health literacy is associated with the effectiveness of the use of preventive and other health services and has consequences for the subjective health status and the mortality of a population [3–6]. Socioeconomic factors such as a low educational level, low social status and migrant background are associated with limited health literacy [7, 8]. Internationally, health literacy has been an important topic in public health research in the past decades. However, in Germany, the biggest country of the European Union, data about health literacy in the general population are still scarce.

International studies have shown that limited health literacy affects large parts of the population [9–11]. According to the European Health Literacy Survey (HLS-EU) almost every second EU citizen had limited health literacy and thus perceived difficulties accessing, understanding and using health information [8, 9]. In Great Britain, more than 50 % showed marginal or low health literacy-skills [7]. Results for health literacy in Germany are scarce. They focus on certain subgroups only [9, 12, 13] or use a short version to measure health literacy [14]. There is also evidence that health literacy declines with increasing age [8]. The decline in health literacy in older age groups is associated with decreasing cognitive functionality and potential health impairments [15, 16].

Nevertheless, adequate health literacy and knowledge about associated factors are relevant in all phases of the life course in order to maintain health and getting involved in decisions about health and health care. However, to date perceived health literacy has not been sufficiently addressed among different age groups and data about factors associated with limited health literacy stratified by different age groups is scarce, especially for middle-aged adults. The aim of this study is therefore to fill this gap and provide data on perceived health literacy stratified for different age groups and analysing its relation to possible determinants such as socio-economic factors or doctor visits in Germany for the first time.

## Methods

### Study population and design

For these analyses, data of the Health Literacy Survey Germany (HLS-GER) were used. In total, 2,000 respondents aged 15 years or older were included in the representative sample in July and August 2014. 258 sample points were randomly selected from a total of 53,000 across Germany, each containing about 700 households. For each sample point, a starting address was selected at random and every third household was selected by a random-walk procedure excluding the starting address. In each of these households, the person who had the most recent birthday was selected. Computer-assisted personal interviews (CAPI) were conducted in German language. The mean interview duration was 53 min. The response rate was 64.9 %.

## Measures

### Health literacy

Health literacy is based on a multidimensional concept taking into account the self-perceived difficulty to perform health information tasks. It was assessed by the HLS-EU-Q-47 questionnaire [17]. Respondents were asked to rate the perceived difficulty of various aspects concerning accessing, understanding, appraising, and applying health information. Item examples are: on a scale from very easy to very difficult, how easy would you say it is to find information about symptoms of illnesses that concern you or to judge which health screenings you should have [8]. In total, the questionnaire comprises 47 items. The degree of difficulty was assessed on a four-point Likert scale from very easy to very difficult. The health literacy score was calculated for respondents with at least 80 % valid items of perceived health literacy. The index was transformed as recommended by the European Health Literacy Project using the following formula:

$$ \operatorname{I}=\left(\operatorname{X}-1\right)\ast \frac{50}{3} $$

The health literacy index ranges from 1 to 50; higher values indicate better perceived health literacy. Internal consistency of the instrument has shown to be good (Cronbach’s alpha: 0.97).

For the analyses, different levels of perceived health literacy were defined as recommended by the HLS-EU-consortium [8]. A health literacy index of 0 to 25 was defined as ‘inadequate’ perceived health literacy, values from > 25 to 33 points as ‘problematic’. Further, health literacy scores of > 33 to 42 were defined as ‘sufficient’, and the remaining interval (> 42 to 50) as ‘excellent’ perceived health literacy. Questionnaire development and criteria for thresholds are described in detail elsewhere [8, 17].

### Independent variables

Social status, gender, education, migrant background, functional health literacy and frequency of doctor visits were included in the analyses as socio-demographic covariates.

Following the HLS-EU [17] functional health literacy was defined as basic objective numeracy and literacy skills in a health-related context in this study. It was operationalized by the Newest Vital Sign Test measuring the ability to read and apply information from an ice cream nutrition label. It comprises six questions testing numeracy and literacy skills [18]. The test was developed and validated in English and Spanish [18]. For this study the validated UK-Version was used [19]. A score of 0 to 3 was defined as limited functional health literacy level while a score of 4 to 6 was categorized as adequate functional health literacy. The UK-Version of the NVS was translated by two independent professional translators into German and then verified in a panel with the German speaking research team of the HLS-EU, the HLS-EU Survey Coordinator, the translators and other relevant health professionals [17]. Face validity and cognitive pre-tests for less educated young people, older people and migrants were conducted in a previous German study [20]. Internal consistency of the German Version of the NVS in this study was acceptable (Cronbach’s alpha 0.73) and comparable to the original UK-Version (Cronbach’s alpha 0.74) [19].

Social status was assessed using a 10-point scale ranging from 1 (lowest position in society) to 10 (highest position in society) [8]. An index of 1 to 4 was defined as low social status; values from 5 to 7 were categorized as medium social status; a score greater than 8 was defined as high social status.

Gender was categorized as ‘female’ or ‘male’.

Educational level was assessed using the International Standard Classification of Education (ISCED-97) [21], which allows for cross-national comparisons of educational levels [22]. ISCED classifies seven levels of educational training, including vocational training. A detailed description of the levels is given by Schneider and Kogan [22]. For the present analysis, educational level was categorized into three groups. Low educational level comprises ISCED levels 0 to 2. This covers education up to lower secondary stage, which often coincides with the end of compulsory education. Medium educational level comprises ISCED levels 3 and 4, which covers upper secondary (level 3) and post-secondary (level 4) education. High educational level comprises ISCED levels 5 and 6, both of which describe tertiary education [22].

Migrant background was assessed by the respondents own and parental country of birth. Respondents born abroad (first generation) or with at least one parent born abroad (second generation) were categorized as having a migrant background. Respondents born in Germany, and whose parents both also were born in Germany, were categorized as not having a migrant background. This conceptualization of migrant background reflects to some extent a person’s personal, cultural and language background regardless of their citizenship.

Frequency of doctor visits was assessed as the number of contacts with a general practitioner during the last 12 months. The answers were categorized into 0 to 2, 3 to 5 and 6 or more contacts.

### Age

Age was recorded in years and categorized into four age groups for the analyses. The youngest age group comprised individuals aged 15 to 29 years and is referred to as ‘adolescents’ given that they are in transition into consumer role, political role, labour force, and having an own family and thus gradually becoming financially and emotionally independent [23]. Individuals aged 30 to 45 years represent a population group with increasing obligations in family organisation, labour market and political and civil engagement. They are labelled as ‘young adults’. Respondents between 46 and 64 years were grouped into ‘middle-aged adults’. Their status can be defined by complex obligations, stabilisation of life plans and saturation of growth [24]. Individuals aged 65 years and older represent the seniors. Most of them are in retirement, face a stepwise reduction of opportunities and physical possibilities and experience at least some severe problems of health management.

### Statistical analyses

Data were analysed using SPSS 23.0. The data were weighted by using Iterative Proportional Fitting to be representative for age, sex, and federal state as compared to the German Microcensus [25]. Descriptive analyses were performed to characterize the study population (Table 1) and describe the distribution of levels of health literacy and scores stratified by age groups. One-way ANOVA was calculated for mean differences between age groups (Table 2). For further analyses, inadequate and problematic levels of health literacy were categorized as ‘limited health literacy’. The association of the covariates with limited health literacy was assessed for each of the different age groups using chi-square tests (Table 3) and multivariate logistic regression (Table 4). There was no multicollinearity between covariates in any of the models.

**Table 1:** Study population of the HLS-GER (*n* = 2,000)

	%	*(n)*
Age		
Mean (SD)	48.2	(18.2)
15–29	19.7	(394)
30–45	24.9	(499)
46–64	31.6	(631)
65–99	23.8	(476)
Gender		
Male	48.9	(977)
Female	51.1	(1,022)
Educational level		
Low	33.6	(669)
Medium	48.9	(972)
High	17.5	(349)
Migrant background		
No	92.1	(1,836)
Yes	7.9	(158)
Functional health literacy		
Limited	19.6	(392)
Adequate	80.4	(1,680)
Social status		
Low	12.9	(252)
Medium	68.6	(1,337)
High	18.5	(360)
Doctor visits		
0–2	56.2	(1,122)
3–5	27.1	(542)
6 or more	16.7	(333)

**Table 2:** Health literacy* scores and levels stratified by age groups

		15–29		30–45		46–64		65–99	
		%	*(n)*	%	*(n)*	%	*(n)*	%	*(n)*
Limited	Inadequate	6.8	(25)	7.0	(34)	9.4	(58)	15.2	(70)
	Problematic	40.5	(152)	40.2	(197)	45.8	(283)	51.1	(236)
Not limited	Sufficient	42.5	(159)	44.3	(217)	37.1	(229)	30.7	(142)
	Excellent	10.3	(39)	8.5	(42)	7.8	(48)	3.0	(14)
Mean**	(SD)	33.8	(6.3)	34.0	(6.0)	32.8	(6.1)	30.7	(6.0)

*measured as perceived difficulty to perform health information tasks

**p-value from one-way ANOVA: *p* <0.001

**Table 3:** Factors associated with limited health literacy* stratified by age groups - results of bivariate analyses

	15–29			30–45			46–64			65–99		
	%	*(n)*	*p*-value**	%	*(n)*	*p*-value**	%	*(n)*	*p*-value**	%	*(n)*	*p*-value**
Gender												
Male	45.5	(95)	0.413	46.5	(104)	0.771	55.2	(159)	0.989	63.7	(145)	0.250
Female	49.6	(83)		47.8	(127)		55.2	(182)		68.9	(162)	
Educational level												
Low	51.2	(78)	0.362	58.1	(72)	**0.019**	65.8	(120)	**0.003**	70.2	(133)	0.254
Medium	43.2	(69)		43.4	(118)		50.2	(159)		65.3	(131)	
High	49.7	(29)		43.9	(42)		53.4	(62)		59.3	(41)	
Migrant background												
Yes	62.9	(24)	**0.039**	72.1	(26)	**0.001**	73.2	(33)	**0.011**	73.7	(26)	0.307
No	45.5	(154)		44.8	(201)		53.8	(309)		65.7	(281)	
Functional health literacy												
Limited	66.9	(36)	**0.002**	60.6	(40)	**0.018**	69.7	(85)	**<0.001**	78.9	(102)	**<0.001**
Adequate	44.0	(141)		45.1	(191)		51.6	(257)		61.5	(205)	
Social status												
Low	68.9	(33)	**0.002**	82.3	(34)	**<0.001**	78.3	(56)	**<0.001**	81.5	(71)	**<0.001**
Medium	47.0	(103)		48.0	(159)		55.4	(241)		65.5	(209)	
High	37.4	(37)		33.4	(36)		38.3	(38)		46.7	(24)	
Doctor visits												
0–2	43.0	(127)	**0.008**	37.8	(135)	**<0.001**	44.2	(143)	**<0.001**	51.0	(57)	**<0.001**
3–5	62.1	(42)		75.2	(67)		64.1	(117)		68.9	(133)	
6 or more	68.0	(8)		66.5	(29)		72.9	(81)		74.1	(116)	
Total	47.3	(178)		47.2	(231)		55.2	(341)		66.4	(307)	

*measured as perceived difficulty to perform health information tasks

**p-values from chi-square tests, significant differences are printed in bold (*p*<0.05)

**Table 4:** Factors associated with limited health literacy* stratified by age groups – results of the multivariate logistic regression**

	15–29			30–45			46–64			65–99		
	OR	95 % CI	*p*-value	OR	95 % CI	*p*-value	OR	95 % CI	*p*-value	OR	95 % CI	*p*-value
Gender												
Male	0.87	0.56–1.34	0.518	1.12	0.74–1.68	0.602	1.11	0.78–1.58	0.562	0.96	0.61–1.50	0.861
Female	ref											
Educational level												
Low	1.05	0.55–2.01	0.872	1.40	0.75–2.62	0.293	1.55	0.92–2.61	0.101	1.16	0.60–2.27	0.661
Medium	0.72	0.38–1.36	0.309	1.14	0.66–1.97	0.631	0.83	0.52–1.32	0.424	0.91	0.49–1.71	0.776
High	ref											
Migrant background												
Yes	1.79	0.84–3.82	0.133	3.22	1.43–7.24	**0.005**	2.14	1.03–4.47	**0.042**	1.12	0.46–2.74	0.798
No	ref											
Functional health literacy												
Limited	1.84	0.94–3.60	0.074	1.87	1.00–3.49	**0.050**	2.01	1.27–3.16	**0.003**	2.25	1.35–3.75	**0.002**
Adequate	ref											
Social status												
Low	3.08	1.43–6.63	**0.004**	9.31	3.56–24.34	**<0.001**	4.61	2.22–9.57	**<0.001**	4.97	2.16–11.43	**<0.001**
Medium	1.27	0.76–2.13	0.355	1.90	1.15–3.16	**0.013**	1.68	1.05–2.70	**0.030**	2.28	1.19–4.38	**0.014**
High	ref											
Doctor visits												
0–2	ref											
2–5	2.14	1.22–3.75	**0.008**	5.51	3.14–9.66	**<0.001**	2.11	1.42–3.14	**<0.001**	2.51	1.51–4.18	**<0.001**
6 or more	2.23	0.60–8.24	0.229	3.83	1.83–8.03	**<0.001**	3.44	2.07–5.73	**<0.001**	3.22	1.85–5.60	**<0.001**

OR > 1 means increased likelihood of having limited health literacy, significant differences are printed in bold (*p*<0.05)

*measured as perceived difficulty to perform health information tasks

**adjusted for all variables in the models

## Results

The mean age of the respondents was 48.2 years. In terms of the distribution of age groups, 19.7 % were adolescents, 24.9 % were young adults, 31.6 % were middle-aged adults, and 23.8 % were seniors. All sample characteristics are displayed in Table 1.

Health literacy scores could be calculated for 1,946 respondents. While adolescents have an average health literacy score of 33.8 and young adults of 34.0, middle-aged adults have a mean score of 32.8 and seniors of 30.7. Scores and levels of perceived health literacy decrease with increasing age (Table 2). There is great variation in health literacy levels between age groups. While 6.8 % of the adolescents and 7.0 % of the young adults were classified having inadequate perceived health literacy, 9.4 % of the middle-aged adults and 15.2 % of the seniors were placed in this category. Only 3.0 % of the seniors were classified as having excellent perceived health literacy, compared to 10.3 % of the adolescents (Table 2).

Limited perceived health literacy was found among 47.3 % of the adolescents and 47.2 % of the young adults. Furthermore, 55.2 % of the middle-aged adults and 66.4 % of the seniors had limited perceived health literacy (Table 3).

Among all age groups, limited functional health literacy, low social status, a migrant background and a high number of doctor visits were associated with limited perceived health literacy. For example, 67 % of the adolescents with limited functional health literacy had limited perceived health literacy compared to 44 % of those with adequate functional health literacy. Furthermore, 69 % of the adolescents with low social status and 37 % of those with high social status were of limited perceived health literacy. Among the subsample of adolescents with migrant background, 63 % were limited in their perceived health literacy, whereas this was the case for 46 % of those without migrant background. Similarly, more than 60 % of those with more than two doctor visits had limited perceived health literacy compared to 43% of the adolescents with a maximum of two doctor visits.

Education was associated with limited perceived health literacy only among adults. No statistically significant relation of gender and limited perceived health literacy could be observed in the bivariate analyses (Table 3).

More than two doctor visits and low social status were statistically significantly associated with limited perceived health literacy among all age groups in the multivariate model (Table 4). Limited functional health literacy was associated with limited perceived health literacy among adults and seniors in the adjusted model. Migrant background was associated with limited perceived health literacy only among adults. Taking other socio-demographic determinants, functional health literacy and doctor visits into account, there was no statistically significant effect of education on limited perceived health literacy in any of the age groups (Table 4).

## Discussion

This study describes perceived health literacy and socio-demographic factors associated with it stratified by different age groups among a representative sample in Germany for the first time.

The most important finding of this study is the different proportion of limited perceived health literacy among the four age groups representing groups of the population in various stages of their life course in Germany, which is in line with previous international studies [7, 15, 26]. Adolescents and young adults have higher levels of perceived health literacy compared to the two older age groups. Anyhow, almost half of the adolescents and young adults in our study – and thus in an early phase of their life – possess limited perceived health literacy. This is of special importance with regard to findings reporting an association between limited health literacy and lower use of preventive health services [27, 28].

Furthermore, our results show that there are subgroups among each age group having high proportions of limited perceived health literacy. For example, our study shows that, almost 70 % of adolescents with low social status have limited perceived health literacy. Among young adults, more than 70 % of those with a high number of doctor visits are also at special risk of having limited perceived health literacy. A high proportion of persons with migrant background also has limited perceived health literacy. Among middle-aged adults, for example, almost 75 % of those with migrant background are limited in their perceived health literacy. Functional health literacy skills play an important role especially among seniors. About 80 % of those with limited functional health literacy skills have a low perceived health literacy. Thus, our study indicates that levels of perceived health literacy are highly diverse among different subgroups in the same phase of the life course. The persistent relation of perceived health literacy with functional health literacy and social status has been indicated by previous research [8, 29] but not described in detail for different age groups.

Our survey holds an interesting detail: Having more than two doctor visits in the last 12 months is statistically significantly associated with limited perceived health literacy among all age groups. A possible explanation for this might be that persons being confronted with decisions about health and health care perceived more difficulties in information tasks and thus show lower levels of perceived health literacy than persons hypothetically thinking about potential difficulties in health information processing but not having been confronted with such situations. Another reason for this could be that persons with limited perceived health literacy more often seek help from their doctor, as they are uncertain or have lower self-efficacy or external locus of control. In addition, the result can be confounded by (perceived) health status, which could not be included in our analyses but is known to be related to health literacy [3–6]. Interestingly, the odds to have limited perceived health literacy when having more than two doctor visits was highest among young adults. This might be explained by an increasing number and complexity of health problems at this age [30] that is typically associated with many different life course decisions in the family, work and community area (‘rush hour of life’).

We did not find a statistically significant effect of education on limited perceived health literacy when also considering functional health literacy. This is not surprising, as functional health literacy subsumes current health related numeracy and literacy skills instead of unspecific skills gained through formal (vocational) education possibly acquired decades ago. Therefore, the ability to use education measured by functional health literacy may overpower the effect of education alone.

We found an association of migrant background with limited perceived health literacy, but only among adults. However, among adolescents and seniors we did not find an effect. An explanation could be that adolescents with migrant background are mostly second-generation and thus differ little from autochthonous young people concerning their general literacy and German language skills [25, 31]. Persons with migrant background in the other age groups may have lower proficiency in German language and therefore use of health information might be perceived more difficult. In addition, their expectations towards information and communication [31] and their skills regarding this might be different from the autochthonous population. An explanation for not showing a relation of migrant background and limited perceived health literacy among the subsample of seniors might be that respondents with migrant background in the oldest age group in our study may not have been representative according their language skills Especially older persons with migrant background with poor German language skills may have been underrepresented as they are difficult to recruit for research [32, 33]. Another reason could be that they have been living in Germany much longer and thus have better German language skills and assimilated to the health care system. However, we could not include any variable verifying this, such as time since immigration, language proficiency or scientific or media literacy. The differences in perceived health literacy among the subgroups may also be explained by the use of different information resources and its reliability and clarity. Persons with migrant background, for example, are known to rely on doctors and family members when seeking for health information and making decisions about health and health care [31]. Higher social class, not having a migrant background and high frequency of health care service use are for example associated with using the Internet for health-related matters [34].

### Strengths and limitations

The present study is the first representative survey in German households. Overall, our study shows a higher proportion of limited perceived health literacy compared to European average [8] which might be explained by different demands and complexity of the health care systems. Our study shows a higher proportion of limited perceived health literacy than other findings from Germany [8, 12, 14]. However, these studies were restricted to the German state of North Rhine-Westphalia, a single region that is not representative for Germany [8, 9], or only used a short version HLS-EU questionnaire to measure health literacy which comprises only a selection of items of the full version [12, 14]. Other studies were restricted to people with statutory health insurance [13], or older populations [12] only.

A strength of this study is the use of the full version of the HLS-EU-Q-47 questionnaire and its performance by computer-assisted personal interviews, which might explain lower health literacy results compared to other studies using pen and paper survey methods [8]. The face-to-face interview situation used in the present study even enabled persons with inadequate reading abilities to take part. Furthermore, the HLS-EU-Q-47 instrument is a subjective self-report measurement tool reflecting perceived health literacy and designed to assess widespread competencies in dealing with health information. Thus, the results do not reflect functional health literacy. However, considering this important concept a measure of functional health literacy was included in the analyses. However, there are also certain limitations associated with the present study. The necessary dichotomization of health literacy certainly leads to loss of precision in calculations. The numbers in the subsamples among the different age groups (e.g. persons with migrant background) are small and our results do not allow drawing definite conclusions. However, this study gives interesting insights into subgroups and topic relevant for further investigation. The number of doctor visits and social status in our study are self-reported and therefore might be imprecise and underlie social desirability. However, perceived measures are important as they represent the user perspective on a topic. Due to the cross-sectional design, it is not possible to determine whether health literacy decreases with age or whether cohort effects such as the use of different information resources affect the health literacy level in different age groups.

## Conclusions

Our results suggest that there is a need to further investigate perceived health literacy in all phases of the life-course. In these age-specific studies, special attention needs to be devoted to subgroups such as people with low social status, limited functional health literacy and/or a high number of doctor visits.

Further research is also needed taking into account the association of different information habits and resources as well as psychological concepts as health literacy was measured as a self-assessed measure. The relation of doctor visits and perceived health literacy should be further assessed considering objective measurements of the frequency of doctor visits and including different types of contacts to the health care system. There is also need for further investigation of factors relevant among persons with migrant background such as duration of stay, language proficiency and information culture. Our findings suggest that it is important to take into account age-specific differences in health literacy in future research. Instruments to measure perceived health literacy should be evaluated among different age groups and adapted accordingly.

## Abbreviations

95 % CI: 95 % Confidence interval
ANOVA: Analysis of variance
CAPI: Computer-assisted personal interviews
EU: European Union
HLS-EU: European Health Literacy Survey
HLS-EU-Q-47: Long version of the European Health Literacy Questionnaire
HLS-GER: Health Literacy Survey Germany
ISCED: International Standard Classification of Education
OR: Odds ratio
SD: Standard deviation
